# Modified Simple Decompression of Ulnar Nerve in Treatment of Cubital Tunnel Syndrome

**Published:** 2012-01

**Authors:** Jamal Gousheh, Ehsan Arasteh

**Affiliations:** Department of Plastic and Reconstructive Surgery, Shahid Beheshti University of Medical Sciences, Tehran, Iran.

**Keywords:** Ulnar nerve, Cubital tunnel syndrome, Simple decompression, Anterior transposition, Inter-muscular septum

## Abstract

**BACKGROUND:**

There are two main surgical approaches, simple ulnar nerve decompression at the elbow, and anterior transposition of the nerve in treatment of cubital tunnel syndrome. Both techniques were reported in literature in details with similar reported success rates. Here, we present a modified simple decompression surgical technique in treatment of cubital tunnel syndrome.

**METHODS:**

Fifty eight patients diagnosed with cubital tunnel syndrome undergoing the presented technique were enrolled. This procedure consisted of ulnar nerve decompression at the elbow and a supplementary procedure of inter-muscular septum transverse cut between triceps and brachialis muscle above the elbow.

**RESULTS:**

Complete sensory recovery was observed in 35 (60.3%) patients, however, mild and occasional sensory symptoms remained in 15 (25.9%), and moderate symptoms persisted in 6 (10.3%) patients. In two patients (3.4%), no sensory improvement was recorded. Post-operatively, muscular hypotrophy improved completely in 5 out of 12 patients (41.7%). However, in the remaining 7 patients (58.3%) with muscular atrophy, motor recovery never took place.

**CONCLUSION:**

The presented modified simple decompression technique was shown to be an effective and safe procedure for the treatment of cubital tunnel syndrome without any complications.

## INTRODUCTION

Compression of the ulnar nerve in the cubital tunnel is the second most common peripheral nerve entrapment syndrome in the upper extremity after carpal tunnel syndrome.^1^^,^^2^ Various etiologies and pathologic conditions, such as trauma, occupational or iatrogenic injuries during general anesthesia, congenital anomalies like cubitus valgus, degenerative or inflammatory diseases, metabolic or nutritional disorders, ganglia, bony impingement, irregularities in muscles and subluxation of the ulnar nerve over the medial epicondyle have been previously described.^3^^-^^5^

The most common presenting symptoms include numbness in the ring and little fingers, weakness of grip, hand clumsiness, atrophy of the hand muscles, and elbow discomfort. In most patients, sensory complaints precede motor deficits. Electrophysiology is considered invaluable to rule out other possibilities in the differential diagnosis.^5^^,^^6^

Treatment modalities include non-operative management and operative procedures. Non-operative treatment including a trial of anti-inflammatory medication, elbow use modification, and soft splinting that were shown to be effective in many early cases.^1^^,^^5^ Operative treatment is indicated for patients with failed conservative treatment or neurological deficits at the 6 week follow up visit.^5^^,^^7^ Surgical methods are divided into two major categories. The first category includes simple ulnar nerve decompression with or without medial epicondylectomy,^8^^-^^12^ and the second includes subcutaneous, intermuscular and submuscular anterior transposition procedures.^1^^,^^5^^,^^13^^-^^15^ However, the optimal surgical treatment still remains controversial and similar success rates have been reported with different surgical techniques.^16^^-^^19^ The method in this study was the combination of the conventional surgical release of the Osborne’s ligament for decompression of the ulnar nerve plus a complementary procedure consisting of the release of the intermuscular septum.

Simple decompression yields good to excellent results with fewer post-operative complications. This technique also does not compromise the vascularity of the ulnar nerve. Therefore, simple ulnar nerve decompression is our preferred surgical method in patients with cubital tunnel syndrome. All patients operated in this study underwent release of the septum between triceps and brachialis muscles above the elbow as an additional procedure. Release of the septum reduced compression on the ulnar nerve when elbow was in flexion. With our experience, this approach yielded satisfactory results without the need to perform additional procedures such as anterior transposition. We performed anterior transposition only in exceptional cases with complicated elbow fractures. 

## MATERIALS AND METHODS

During 2001-2008, 61 patients with cubital tunnel syndrome underwent operation. Three patients with severe elbow deformity due to fracture leading to ulnar nerve entrapment by callus formation were excluded from the study. These three patients underwent sub-muscular transposition to move the ulnar nerve to an undamaged area. The remaining 58 patients underwent simple decompression and intermuscular septotomy. This group of patients included nine (15.5%) women and 49 (84.5%) men with the average age of 38 (range=29–60) years. The patients complained of numbness or tingling in the ring and little fingers, hand weakness, and pain along the ulnar border of the hand and forearm. Preoperative motor deficits were found in 12 (20.7%) patients. 

Standard radiography of elbow was performed for all patients. Radiography was helpful in diagnosing severe elbow deformities due to old fractures. Preoperative electrodiagnostic examination was also performed in all patients. Ulnar nerve compression in the cubital tunnel was confirmed in all 58 patients and other differential diagnoses were ruled out. 

**Fig. 1:**
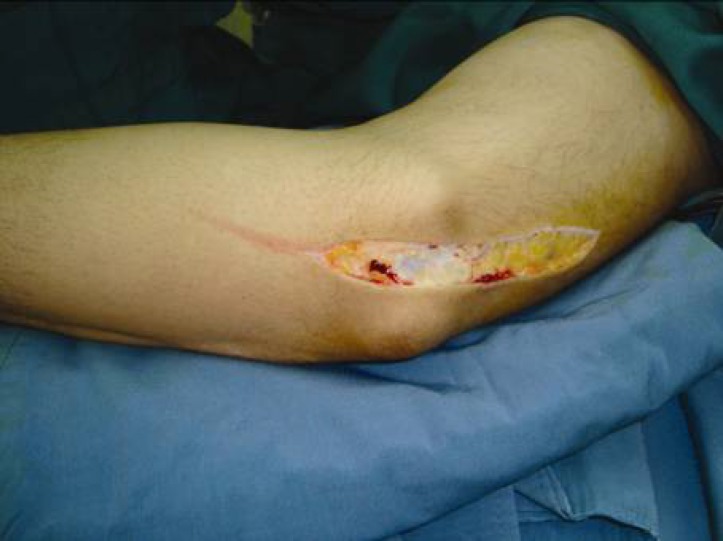
With the elbow flexed at 90 degrees, an 8 to 10 cm curved skin incision above and below the elbow is made posterior to the medial epicondyl.

**Fig. 2:**
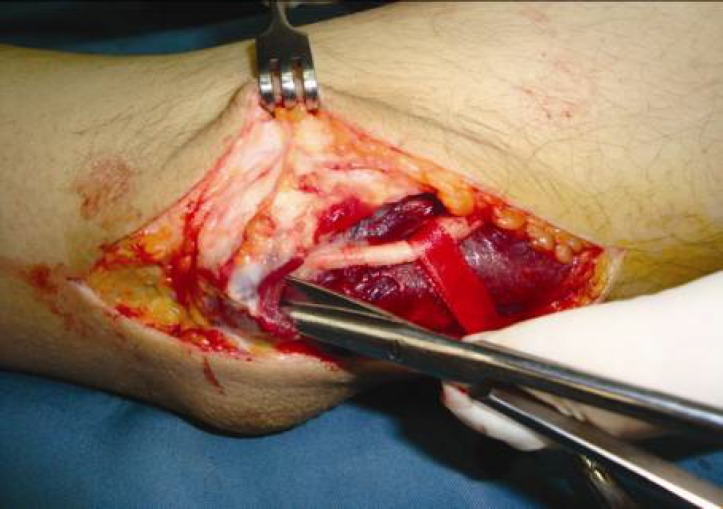
The ulnar nerve is released proximal and distal to the elbow.

**Fig. 3:**
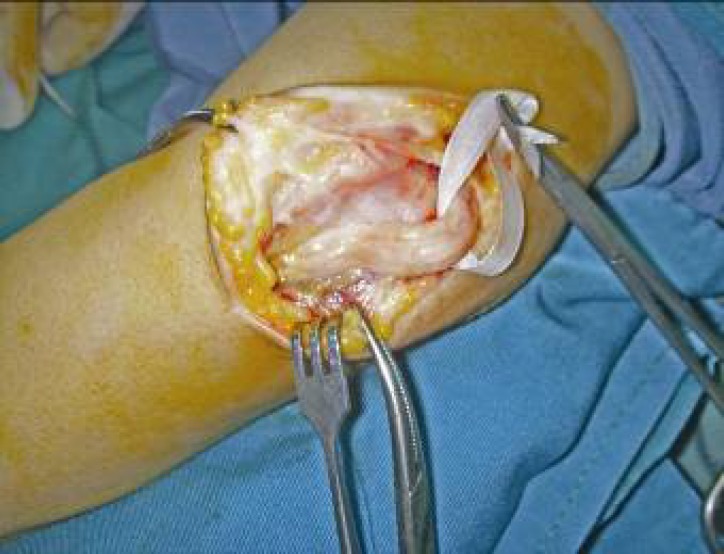
Obvious compressive effect on the ulnar nerve is observed after Osborn ligament release

**Fig. 4:**
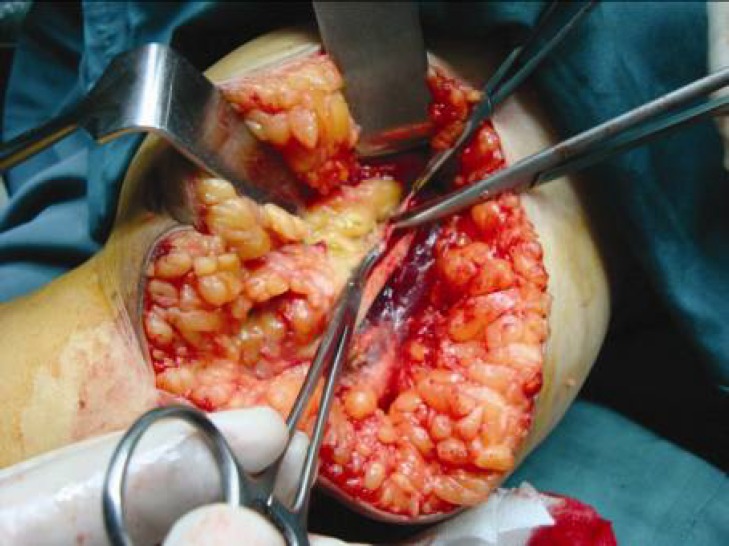
The inter-muscular septum between triceps and brachialis muscles which is parallel to the ulnar nerve is cut transversally 2 to 3 cm above the elbow. This supplementary technique leads to elongation of the cubital tunnel and reduce compression on the ulnar nerve during elbow flexion.

The operative procedure was performed under general or axillary anesthesia and applying a pneumatic tourniquet. The elbow was flexed to 90° and the arm was externally rotated. An 8 to 10 cm curved skin incision above and below the elbow was made posterior to the medial epicondyle (Figure1). The ulnar nerve was released proximally along the medial intermuscular septum. The cubital tunnel retinaculum and flexor carpi ulnaris aponeurosis (Osborne ligament) were then cut distally to release the ulnar nerve (Figure2). Osborne ligamentous band which was connected to heads of FCU muscle was cut 4 to 7 centimeters distally. Usually, a circular effect of compression over the ulnar nerve could be observed under the upper head of the flexor carpi ulnaris muscle (Figure 3). It is important to keep the ulnar nerve connected to its accompanied vessels in its primary position and avoid circumferential dissection around the nerve. Next, the inter-muscular septum between triceps and brachialis muscles which was parallel to the ulnar nerve was cut transversally 2 to 3 cm above the elbow (Figure 4). Finally, after homeostasis; the wound was closed. Early rehabilitation including light flexion and extension of the elbow to 20° and wrist and finger movements were encouraged. Two weeks postoperatively, free movement in the entire extremity was recommended.

## RESULTS

Symptoms including numbness and tingling were improved in all patients the day after the operation. Final outcome of the procedure was evaluated 2 to 4 months post-operatively. Sensory deficits disappeared completely and the patient became asymptomatic in 35 (60.3%) cases. However, mild and occasional sensory symptoms remained in 15 (25.9%), and moderate symptoms persisted in 6 (10.3%) patients. No post-operative improvement was observed in two (3.4%) patients. These two patients were re-operated later by other surgeons with the diagnosis of thoracic outlet syndrome; however, the secondary operation did not improve the patients’ symptoms, and they complained of vague pains. Twelve (20.7%) patients had obvious muscular hypotrophy in the territory of the ulnar nerve. Post-operatively, muscular hypotrophy improved completely in 5 out of 12 patients (41.7%). However, in the remaining 7 patients (58.3%) with muscular atrophy for more than a year, although sensory improvement was achieved, motor recovery never took place. 

## DISCUSSION

The optimal surgical treatment for compressive neuropathy of the ulnar nerve at the elbow is controversial. Numerous studies have not shown any definitely superior procedure among the surgical options such as simple decompression and anterior transposition of the ulnar nerve.^1^^,^^8^^,^^20^^,^^21^ However, many authors have reported that simple decompression was the choice procedure for the majority of cases with cubital tunnel syndrome.^9^^,^^10^^,^^21^^,^^22^ Some authors also mentioned that anterior transposition of the ulnar nerve might often be harmful and had potential complications.^8^^,^^23^

As many other authors discussed, when all surgical techniques yield similar success rate, the simpler one should be chosen.^8^^-^^10^ Simple decompression is technically simple and does not compromise the vascularity of the nerve. It is reasonably effective with a satisfactory outcome from 80% to 90% of patients in various studies.^7^^,^^24^ Simple decompression only targets the cubital tunnel, the primary site of compression, which is usually observed under the upper head of the flexor carpi ulnaris muscle as an obvious compressive effect on the nerve. Therefore, this technique has a lower rate of post-operative complications enabling patients to start early rehabilitation program. Simple decompression, however, is not appropriate in few exceptional conditions such as, poor bed, severe cubitus valgus and complicated fractures of the elbow.^3^^,^^8^^,^^25^

Intermuscular septum between triceps and brachialis muscles above the cubital tunnel was cut in this series of patients as a modification to the simple decompression of the ulnar nerve. This supplementary technique led to elongation of the cubital tunnel and reduced compression on the ulnar nerve during elbow flexion without the added post-operative complication.

Many surgical procedures and modifications were presented for the treatment of cubital tunnel syndrome. However due to similar success rates, the dilemma about the choice and superior method has not yet been resolved. The results in this study group showed that the presented modified simple decompression technique has been an effective and safe procedure without unnecessary complications.

## CONFLICT OF INTEREST

The authors declare no conflict of interest.
